# Mitogen Activated Protein Kinase (MAPK) Activation, p53, and Autophagy Inhibition Characterize the Severe Acute Respiratory Syndrome Coronavirus 2 (SARS-CoV-2) Spike Protein Induced Neurotoxicity

**DOI:** 10.7759/cureus.32361

**Published:** 2022-12-09

**Authors:** Anthony M Kyriakopoulos, Greg Nigh, Peter A McCullough, Stephanie Seneff

**Affiliations:** 1 Infectious Disease, Nasco AD Biotechnology Laboratory, Piraeus, GRC; 2 Naturopathy, Immersion Health, Portland, USA; 3 Cardiology, Truth for Health Foundation, Tucson, USA; 4 Computer Science and Artificial Intelligence Laboratory, Massachusetts Institute of Technology, Cambridge, USA

**Keywords:** autoimmunity, covid-19, senescence, aging, autophagy, wip1, p53, prion and prion-like diseases, mrna vaccines, sars-cov-2 spike protein

## Abstract

The severe acute respiratory syndrome coronavirus 2 (SARS-CoV-2) spike protein and prions use common pathogenic pathways to induce toxicity in neurons. Infectious prions rapidly activate the p38 mitogen activated protein kinase (MAPK) pathway, and SARS-CoV-2 spike proteins rapidly activate both the p38 MAPK and c-Jun NH2-terminal kinase (JNK) pathways through toll-like receptor signaling, indicating the potential for similar neurotoxicity, causing prion and prion-like disease. In this review, we analyze the roles of autophagy inhibition, molecular mimicry, elevated intracellular p53 levels and reduced Wild-type p53-induced phosphatase 1 (Wip1) and dual-specificity phosphatase (DUSP) expression in neurons in the disease process. The pathways induced by the spike protein via toll-like receptor activation induce both the upregulation of PrP^C^ (the normal isoform of the prion protein, PrP) and the expression of β amyloid. Through the spike-protein-dependent elevation of p53 levels via β amyloid metabolism, increased PrP^C^ expression can lead to PrP misfolding and impaired autophagy, generating prion disease. We conclude that, according to the age of the spike protein-exposed patient and the state of their cellular autophagy activity, excess sustained activity of p53 in neurons may be a catalytic factor in neurodegeneration. An autoimmune reaction via molecular mimicry likely also contributes to neurological symptoms. Overall results suggest that neurodegeneration is in part due to the intensity and duration of spike protein exposure, patient advanced age, cellular autophagy activity, and activation, function and regulation of p53. Finally, the neurologically damaging effects can be cumulatively spike-protein dependent, whether exposure is by natural infection or, more substantially, by repeated mRNA vaccination.

## Introduction and background

A significant percentage of patients infected with severe acute respiratory syndrome coronavirus 2 (SARS-CoV-2) develop neurological and cognitive impairments, sometimes lasting long after the infection has cleared. This condition has been named “long haul COVID disease,” or simply “long COVID,” also known as “PASC” (post-acute sequelae of SARS-CoV-2 infection). An international study quantified persistent long-COVID symptoms among 3,762 individuals following a SARS-CoV-2 infection. Memory and cognitive dysfunction were experienced in over 88% of the respondents. These were the most persistently observed neurological symptoms, and they were equally common across all ages. These disabilities substantially impacted the subjects' ability to carry out their work and daily life activities. Both central and peripheral nervous system damage was implicated [1]. A post-mortem study of the brains of three patients who died from severe coronavirus disease 2019 (COVID-19) showed a large number of activated microglia that were associated with overexpression of inflammatory markers, including interleukin-1β (IL-1β) and IL-6. The authors suggested that oxidative stress induced a glial-mediated neuroinflammatory response leading to neuronal injury [2].

A growing consensus attributes these symptoms to the neurotoxic effects of the spike glycoprotein, particularly the S1 subunit [3]. A paper published by Idrees and Kumar showed that the receptor-binding domain of the SARS-CoV-2 spike S1 protein binds to heparin and to heparin-binding proteins. These authors proposed in their conclusion that the stable binding of the S1 protein to these proteins might initiate the aggregation of brain proteins and accelerate neurodegeneration [4]. A study evaluating the amyloidogenic potential of the spike protein verified that the spike protein can cause amyloid-like fibrils to appear after the protein has been subjected to proteolysis. A specific segment that appeared following proteolysis, spike 194-213 (FKNIDGYFKI), was demonstrated both theoretically and experimentally to be amyloidogenic [5]. A paper by Castelletto and Hamley focused on another specific sequence in the SARS-CoV-2 spike protein near the fusion sequence, namely "RSAIEDLLFNKV," which was demonstrated experimentally through spectroscopic analysis to switch from its normal alpha-helical structure to a beta-sheet structure upon changes in pH. The beta-sheet form, prominent at pH 4.4, was amyloidogenic and also supported hydrogel formation [6]. A study by Kruger et al. found that proteolysis-resistant fibrin amyloid microclots accumulate in the blood in association with PASC, and this also suggests that the spike protein has amyloidogenic properties [7].

Direct experimental evidence of the toxic effects of S1 in the brain comes from studies conducted by a team of Korean researchers, published in 2022 [8]. In the experiment, S1 subunits were introduced directly into the dorsal hippocampus of mice, and it was shown that the mice subsequently suffered from anxiety-like behavior and cognitive deficits. Further experiments both in vivo and in vitro found that the effects were mediated by microglia, a special type of macrophage in the central nervous system (CNS). The microglia became activated following exposure and released excitatory cytokines, in particular IL-1β. IL-1β expression was upregulated more than seven-fold in the hippocampi of the exposed mice. Morphologically, the microglia of the exposed mice acquired the features of reactive microglia.

Farsalinos et al. have hypothesized that the toxicity of the spike protein may be partially attributed to an ability to suppress the action of the neurotransmitter acetylcholine. Several known neurotoxins bind to nicotinic acetylcholine receptors and inhibit their function. The authors identified certain sequences in the spike protein that shared similarities with known toxins that bind to these receptors. They even suggested that nicotine could be therapeutic because it stimulates these receptors [9].

In this article, we attempt to trace the likely biological pathways by which neuronal damage occurs in response to the spike protein, particularly S1. Based on the emerging literature, we will argue that toll-like receptor 4 (TLR4) signaling is central to the destructive reaction process. An important intermediary is the MAPK cascade. MAPK comprises four distinct pathways: a) the extracellular signal regulated kinase 1 and 2 (ERK1/2); b) the ERK-big MAP kinase 1 (BMK1); c) the c-Jun NH2-terminal kinases (JNK) or stress activated protein kinases (SAPKs); and d) the p38 MAPKs. The ERK pathways are stimulated by growth factors, hormones, and pro-inflammatory stimuli whereas the JNK and p38 MAPK are activated by cellular and environmental stress signals in addition to pro-inflammatory stimuli [10-11]. It is these latter two pathways that we will argue play a primary role in spike protein neurotoxicity.

Recent neurotoxicity studies indicate that the SARS-CoV-2 S1 subunit induces neuro-inflammation in microglial cells [12-13]. The neuroinflammatory response is mediated by p38 MAPK and nuclear factor κ-light chain enhancer of activated B cells (NF-κB) activation, mainly through the pattern recognition receptor TLR4. In addition, the SARS-CoV-2 S1 subunit elicits a pro-inflammatory response in murine and human macrophages by activating TLR4 receptor signaling. In this signaling process, both JNK and p38 are activated by phosphorylation [14]. It is important to note that infectious prions also activate the p38 MAPK pathway to induce their neurotoxicity effects [15]. The spike protein has prion-like characteristics that may contribute to its neurotoxicity. We will return to these topics in great detail later.

## Review

Special considerations for the mRNA vaccines

All of the COVID vaccines on the market today are based on the principle of inducing the immune cells to produce antibodies to the spike protein. While some vaccines, such as Sinovac, use a more traditional strategy based on an inactivated version of the virus, the three main vaccines marketed in the United States are all based on gene therapy -- a nucleotide sequence that codes for the spike protein gets copied into protein in the cells that take up the vaccine. The Johnson and Johnson vaccine (and also the AstraZeneca vaccine that is commonly used in Europe) is based on an inactivated adenovirus augmented with code for the spike protein inserted into its DNA sequence. Once the virus infects cells, it produces the spike protein using its standard tools for converting its own DNA into RNA and finally into protein.

The two mRNA vaccines widely distributed in the United States, marketed by Moderna and Pfizer/BioNTech, use a novel approach that has never been previously used for any vaccine on the market for any disease. The technology behind these two vaccines is complex and sophisticated, and much about it is new and poorly evaluated for safety [16]. The mRNA in the vaccines is very different from the mRNA sequence that the virus uses to encode the spike protein. A significant modification was to replace all of the uridines in the sequence with methylpseudouridines [17]. This allows the mRNA to resist enzymatic breakdown. It has been shown that methylpseudouridine modifications support more than 10 times as much protein as unmodified mRNAs, in part by preventing repression of translation initiation [18-19].

The modified spike protein mRNA is encapsulated within a highly engineered lipid nanoparticle made up of cholesterol and other phospholipids. Other ingredients in the lipid nanoparticles include polyethylene glycol and a synthetic cationic lipid, which facilitates escape from the lysosome into the cytoplasm and initiation of protein synthesis. The actual sequence itself is also modified, through a process called “codon optimization,” which involves substituting redundant codons that translate more efficiently than the codons the virus used for each amino acid. A codon replacement that actually changes the peptide sequence is also introduced, replacing two adjacent amino acids with a double proline sequence that disrupts the refolding step to facilitate membrane entry following binding to the angiotensin converting enzyme 2 (ACE2) receptor. Finally, the mRNA molecule is “humanized” by inserting 5’ and 3’ untranslated regions (UTRs) on its two ends, sequences borrowed from long-lasting human mRNAs, and adding a long poly-A tail to further promote resistance to breakdown [16]. The spike protein shares regions of high molecular similarity with many important human proteins, and molecular mimicry may lead to autoimmune disease, especially because the vaccine induces a very strong immunoglobulin G (IgG) antibody response [20]. We will return to this topic later in this article.

It appears that the developers of the mRNA vaccines were very successful in assuring rapid synthesis of the spike protein sustained over a long period of time. Most human mRNA molecules are eliminated within a few hours of their synthesis, whereas spike protein mRNA has been found in the draining lymph nodes of the arm muscle two months after vaccination, and this durability was associated with post-vaccine symptoms similar to the symptomatic profile of long COVID [21]. Fertig et al. have found mRNA circulating for at least two weeks after vaccination [22].

While all of the SARS-CoV-2 vaccines are problematic because they introduce the toxic spike protein into the body, we suggest that the mRNA vaccines may be especially dangerous because of their potential to introduce large quantities of spike protein over an extended time.

CD16+ monocytes, microRNAs, and spike protein persistence​​​​​​

Remarkably, the spike protein has been found to persist in human CD16+ monocytes circulating in the blood as much as 15 months after infection with SARS-CoV-2 [23]. Spike persistence was associated with long COVID symptoms, and it was suggested that persistent spike presence could explain lingering symptoms. This was not reflecting an active infection, as only fragmented SARS-CoV-2 RNA was found in these PASC patients. This finding is mysterious, as 15 months seem too long for either a protein or a messenger RNA molecule to survive.

It is possible that this feat is achieved through a process that includes reverse transcription of the viral mRNA coding for the spike protein into DNA [24]. A recent in vitro study on the mRNA in the SARS-CoV-2 vaccines has shown that such a capability exists in human cells. These authors demonstrated that human liver cancer cells are able to convert the mRNA from the COVID vaccines into DNA within six hours of exposure [25]. Cancer cells are known to often express high levels of long interspersed nuclear element-1 (LINE-1), a retrotransposon that is capable of reverse transcribing mRNA into DNA. Expression of LINE-1 is higher in tumors with p53 mutations [26].

Furthermore, and remarkably, tumors release extracellular vesicles containing retroelements that can be taken up by circulating monocytes, especially under inflammatory conditions [27]. This suggests a mechanism by which the CD16+ monocytes could acquire the capability to reverse transcribe mRNA. Alternatively, tumor cells could be releasing exosomes containing mRNA coding for the spike protein, which is then taken up directly by the circulating monocytes and translated into protein [28].

Furthermore, the CD16+ monocytes themselves are likely to be long-lived. The CD16+ subset of circulating monocytes is known as the “inflammatory” subset, because they typically release higher amounts of inflammatory cytokines such as tumor necrosis factor-α (TNF-α), and low levels of the anti-inflammatory cytokine IL-10, in response to toll-like receptor (TLR) stimulation, compared to “classical” CD16+ monocytes. Normally, they make up 10%-20% of the circulating monocyte pool, but their numbers are expanded in association with inflammatory conditions.

MicroRNAs (miRNAs) are short single-stranded non-coding RNA molecules that function in post transcriptional regulation of gene expression. Within the concept of autophagy regulation and its association to disease, the miRNAs have a protagonist role by upregulating and/or downregulating autophagy mechanisms [29]. For example, under the pathological stress conditions of autoimmunity, such as the increased levels of c-Myc transcription factor in Crohn’s disease, specifically the enhanced expression of miR-106B and miR-93 inhibit autophagy by reducing autophagosome formation [30].

Moreover, the dysregulation of expression of a number of miRNAs is implicated in neurodegenerative disease pathogenesis as in the case of Alzheimer’s disease (AD). The miRNA contributions to disease etiopathogenesis are achieved through differential modulation of autophagy [31]. Furthermore, the upregulation of specific miRNAs, as in the case of miR-101a, would reduce autophagic vacuole formation by inhibiting the expression of MAPK-1 [32]. The expression of MAPK-1 is needed to initiate the ERK pathway [10]. Decreased ERK activity is associated with reduced autophagic capability of cells that results in either cell death or senescence [33].

In relevance to this study, miR-146a in particular, is a well-known marker for a “senescent ” phenotype. The basal level of this miRNA is significantly higher in CD16+ monocytes than in the classical monocytes. Senescence is a long-lived state of irreversible proliferative arrest. The senescent monocyte remains alive for an extended period, continually releasing inflammatory cytokines [34].

Another mechanism by which the spike protein could persist long-term would be through its misfolding into a protease-resistant form. The spike protein is a glycoprotein, and glycoproteins from viruses have been shown to facilitate the spreading of proteopathic seeds. In a seminal experiment, researchers clearly demonstrated that the spike protein could facilitate intercellular transfer of exosomes containing cytosolic prions and tau aggregates, accelerating prion-like spreading. Cells propagating tau aggregates were first transfected with a vector coding for the spike protein. Immortalized human embryonic kidney cells (HEK cells) served as recipients of exosomes released by the transfected cells. Transfected donor cells were cocultured with recipient HEK cells overexpressing ACE2. The S1 segment was identified in lysates of the source transfected cells, and also showed up in extracellular vesicles secreted by these cells. The presence of spike protein expression in the source cells resulted in a significant increase in the number of recipient cells with induced aggregates [35].

 In a study investigating the durability of spike protein production following vaccination, abundant spike protein was still present in germinal centers in draining lymph nodes 16 days after the second vaccine, and spike antigen was still present as late as 60 days after the second vaccine [36]. A 2022 study by Bansal et al. showed that the spike protein appeared in circulating exosomes 14 days after the first mRNA vaccine dose, and that spike-containing exosomes were still detectable four months later. They argued that these exosomes played an essential role in the induction of antibodies [37].

TLR4 receptor activation, CD16+ monocytes, and brain inflammation

While it is well established that the SARS-CoV-2 virus gains entry into human cells via the ACE2 receptor, there is another activation pathway that may be responsible for the cytokine storm associated with severe disease. A gene expression assay study involving peripheral blood mononuclear cells drawn from 48 subjects, including 28 COVID-19 patients (8 severe vs. 20 mild) revealed that severe cases were associated with activation of TLR4 signaling and a response that bore a strong resemblance to bacterial sepsis [38]. Furthermore, in vitro studies on both human and mouse macrophages demonstrated that the S1 subunit of the spike protein alone activates TLR4 receptors and induces a strong inflammatory response via the NF-κB and JNK pathways [14]. The spike protein has also been shown to activate TLR2 [39]. This receptor is specifically associated with induction of IL-6 [40]. A case study involved four individuals who died of an "unknown cause" following a second dose of an mRNA vaccine. RNA sequencing revealed that genes involved in neutrophil degranulation and a cytokine storm were sharply upregulated in the cases compared to controls, suggesting that the vaccines induced an excessive inflammatory response [41]. Another experiment showed that the S1 subunit of the SARS-CoV-2 spike protein interacts specifically with the extracellular leucine rich repeat domain of TLR4 to activate NF-κB [42].

The TLR4 is a transmembrane member of the TLR family that is known for its sensitivity to bacterial infections. It is expressed mainly by immune cells of myeloid lineage, and its activation induces the NF-κB inflammatory signaling response, which activates the innate immune system to respond to the infection. The best-known stimulator of the TLR4 response is bacterial lipopolysaccharide (LPS). There is an acidic four-amino-acid sequence (PRRA) in the S1 segment of the spike protein, just above the furin cleavage site, unique among coronaviruses, that is also found in Staph aureus enterotoxin B (SEB), an extremely toxic enterotoxin. SEB is a potent inducer of TNF-α, and it induces an expansion of the pool of CD16+ monocytes. SARS-CoV-2 entry into cells can be inhibited by a monoclonal antibody against SEB [43-44]. It is possible that TLR activation by spike depends in part on this unique sequence.

The CD16+ cells are known for their more mature stage compared to other circulating monocytes. They are the primary cell type that infiltrates inflammatory tissues and launches the TLR4 signaling cascade [45].

When inflammation is occurring outside the CNS, there is a systemic response that takes place in the brain, whereby microglia become activated and upregulate TNF-α signaling. Subsequently, circulating monocytes are recruited into the brain through enhanced expression of cerebral monocyte chemoattractant protein (MCP)-1 [46]. Through such a mechanism, it is possible that CD16+ monocytes deliver spike protein to the brain, causing neuronal injury and explaining cognitive issues linked to long COVID.

The TLR4 activation (e.g., launched by CD16+ cells in the vessel wall) induces T cells to invade the tissues and upregulates expression of the chemokine CCL20, resulting in vasculitis [47]. A case in point involved a 76-year-old man with Parkinson's disease who died three weeks after his third immunization against COVID-19 (the BNT162b2 mRNA vaccine) [48]. Histopathological analyses of the brain revealed acute lymphocytic vasculitis and multifocal necrotizing encephalitis. Immunohistochemistry analysis identified the spike protein but not the nucleocapsid protein in the foci of inflammation in both the brain and the heart. The patient had not been previously diagnosed with COVID-19, so there is strong evidence that the vaccine caused this condition.

Aggregation-prone prion protein: normal function and expression

Central to prion disease pathology are the conformational changes of the normal prion protein (PrP) isoform, PrPC, which is located primarily on the surface of nerve cells. Conformational changes on the tertiary structure of PrPC result in the infectious form of the protein, also referred to as the misfolded isoform PrPSC (SC stands for “scrapie,” the prion disease that occurs in sheep). These misfolded proteins aggregate into long fibrils and deregulate normal functioning of the brain, leading to prion-related disease such as scrapie, Alzheimer's disease (AD), and several others [49]. The non-infectious form PrPC, under non-pathogenic conditions, plays many beneficial cellular roles. It participates in lymphocyte activation, cellular differentiation, neurite outgrowth, synaptogenesis, cellular signaling and viability, cellular adhesion processes and many other important functions for cellular homeostasis [for review see Castle and Gill (2017) [50]].

Overall, PrPC is a stress-induced protein offering cellular protection under stress conditions, and its normal level is increased under conditions of hypoglycemia, ischemia, and in the presence of insulin. Some of the many beneficial roles of PrP and also of β-amyloid precursor protein (APP), which is linked to AD, are presented in Table 1. The expression of PrPC is subject to a plethora of transcription factors that are elevated by stress-inducing cellular conditions. Endoplasmic reticulum stress also induces PrPC expression, as shown in Table 1 [50]. The prion protein gene (PRNP), although it may be regarded as a housekeeping gene, has multiple binding sites for transcription factors in its promoter region, including the selective promoter factors Sp1 and Sp2, normally known for their tumorigenicity potential [51].

**Table 1:** Some of the normal PrP and APP physiological functions. PrP, prion protein; NMDAR, N-methyl-D-aspartate receptor; ER, endoplasmic reticulum; APP, β-amyloid precursor protein

PrP^C^	
Function	Effects and properties
Stress and neuroprotection	Anti-oxidative stress response [52-53] protection from ER-stress induced apoptosis [54]
Regulation of autophagy	Supports autophagy by facilitating autophagosome-lysosomal fusion [55]
Regulation in cancer progression	Induction of cell survival in tumor cells [56]
APP	
Stimulation of cellular growth	Proper neurite outgrowth [57]
Neural stem cells viability	Increases and sustains the proliferation of neural progenitor cells [58-59]
Regulation of synaptic plasticity, learning, and memory	Supports dendritic spine formation during development [60]; enhances NMDAR function [61]
Regulation of blood coagulation and wound repair	Accumulation in platelet α granules and release during wound healing [62] Anti-coagulant properties to regulate thrombosis after cerebral vascular injury [63]

Activating protein-1 (AP-1) and AP-2, along with a variety of dimers of the Jun and Fos family, are among several transcription factors with a high affinity for the GC-rich putative binding and promoter regions within PRNP. Activation by these transcription factors plays a regulatory function in the brain [64]. These transcription factors are operational as a consequence of JNK activation and c-Jun phosphorylation, as well as the E4 promoter binding protein. Expression of this binding protein depends on the intracellular levels of calcium (Ca2+), together with many additional transcription factors involved in phosphatase pathway regulation [65-66].

Interestingly, in experiments that induce the antisense silencing of PrPC expression, the result is phosphorylation of 4E binding protein-1 (4EBP-1), a molecular event that causes the release of eukaryotic translation initiation factor 4E (eIF4E) to proceed to cap-dependent mRNA translation. This in turn causes autophagy-dependent cell death in glioma cells [67-68].

Notably and relevantly, it has been shown that inducing cells to favor cap-dependent translation via the high affinity between caps of synthetic mRNAs and eIF4E drives the recipient cells toward an increased tendency for proliferation and towards initiation of the cellular events that favor oncogenesis, immune dysregulation, and aging defects. The synthetic mRNA cap currently resident on the SARS-CoV-2 mRNA used for genetic vaccination is precisely the cap composition that favors cap-dependent translation of the mRNA. Furthermore, there are at least two additional cellular factors also driving cap-dependent translation in cells stressed by the presence of the synthetic mRNA and its spike protein product. These include a) the p38 MAPK pathway and b) the imbalance of p53 inhibitory activity toward the mechanistic target of the rapamycin (mToR) axis [69].

In summary, the mRNA vaccines currently in use bring about a constellation of circumstances that drive cells toward cap-dependent translation of that mRNA -- a process with a number of expected but not well-characterized detrimental effects on cellular homeostasis.

Prion protein and autophagy

An impairment or failure of macro-autophagy is being increasingly recognized as a primary contributor to prion disease [70-71]. Autophagy can control prion infection through its ability to clear aggregation-prone proteins that would otherwise accumulate in neurons [72]. Macro-autophagy is an important pathway by which misfolded prion protein itself is degraded, and drugs that induce autophagy have been shown to have anti-prion effects [73]. Autophagic vacuoles normally form and then fuse with endolysosomes for eventual clearance [74]. With increased autophagy activity, the neuron is less likely to release prion proteins within exosomes to induce the spread of infectivity to other neurons [73]. Interestingly, the prion protein is upregulated under multiple stressed conditions, and it has been proposed that an important role it plays is to facilitate the fusion of the autophagosomes with lysosomes to promote clearance of cellular debris -- including misfolded proteins and damaged mitochondria.

There exist strains of mice used in research laboratories that have a genetic mutation in the PRNP which disables its expression. These mice provide important knowledge about the functions of the prion protein by virtue of its absence. A key feature of these mice is the appearance very early in the life of autophagic vacuoles in the cytoplasm. Vacuoles appeared as early as three months of age in cortical neurons, and by six months they had also appeared in hippocampal neurons. The number of vacuoles increased in the hippocampus at an accelerated rate with aging compared to control mice. These defective mice were more sensitive to oxidative stress, and they had an increased risk of seizures, motor and cognitive abnormalities, and impaired long-term potentiation in the hippocampus [75]. These mice provide strong support for the view that the prion protein supports autophagic clearance of cellular debris.

Curiously, the accumulation of autophagic vacuoles is also a common feature of neurodegenerative diseases, including Creutzfeldt Jakob Disease (CJD) [71]. The fact that both too little and too much prion protein lead to similar disease states can be explained if we assume that prion disease is mainly a loss-of-function pathology. When the neuron is exposed to stressors that increase the burden of misfolded proteins, it upregulates PrP to assist in the removal of this debris via the lysosomal system. But once there are seed misfolded PrPSc proteins, or externally supplied misfolded prion-like proteins such as the spike protein, along with the high concentration of PrP induced by the stressors, there is the potential for the seed to recruit most of the PrP present in the cytoplasm, converting it first to soluble oligomers and finally to precipitated fibrils. While the amount of PrP in the cell is high, most of it is tied up in the oligomers and fibrils, so it is no longer able to clear the debris, resulting in the accumulation of autophagic vacuoles.

Spike protein, molecular mimicry, and autoimmune disease

It has been known for at least two decades that molecular mimicry can induce autoimmune disease [76]. An analysis of the peptide overlap between the SARS-CoV-2 spike glycoprotein and various mammalian proteomes revealed that, among the species analyzed, only the human, mouse, and rat proteomes had significant overlap, at the hexapeptide and heptapeptide levels. Furthermore, those species with little overlap (cats, dogs, and three other primates) were also not susceptible to symptomatic disease from exposure to SARS-CoV-2. A conclusion was that molecular mimicry may be the primary cause of symptomatic disease [77].

Nunez-Castilla et al. have suggested that autoimmunity due to cross-reacting antibodies could explain several of the symptoms associated with COVID-19, such as thrombocytopenia, platelet activation, calcium dysbiosis, and cardiovascular disease. They singled out a TQLPP motif and an ELDKY motif in the spike protein as especially problematic examples of the potential for cross-reactivity [20].

Vojdani and Kharrazian have performed an experiment to assess the potential for the spike protein to cause autoimmune disease via molecular mimicry. They used commercially available mouse monoclonal antibodies against the SARS-CoV-2 spike protein and assessed their potential to bind to 50 different human tissue antigens. Several proteins that are associated with autoimmune disease were identified as having significant cross-reactivity, including transglutaminase, myelin basic protein, mitochondria, nuclear antigen, α-myosin, thyroid peroxidase, collagen, claudin 5+6, and S100B [78].

Of concern, mRNA COVID vaccine-based immunity has been demonstrated to induce an imbalanced antibody response favoring IgG antibodies over secreted mucosal (IgM) antibodies. In a population study, COVID-19 mRNA vaccines elicited a considerably weaker mucosal antibody response in the lungs than the levels produced in COVID-19 convalescents [79]. This is not surprising, since the vaccine is injected past the mucosal barriers. However, it has been shown that the absence of secreted IgM leads to accelerated development of autoimmune disease [80].

Linear sequence similarity between segments of the spike protein and several proteins involved in maintaining nerve conduction in the nervous system suggests the potential for neurological disease in response to the mRNA vaccines [81]. In particular, there is potential for the spike protein to cause prion-like disease through a mechanism that is based on molecular mimicry. The prion protein plays important although poorly understood role in the endoplasmic reticulum (ER), and its globular C-terminal domain is essential for import into the ER [82]. Remarkably, antibodies specific to the globular domain induce a condition that resembles Creutzfeldt-Jakob disease, only with an accelerated rate of decline [83]. The mechanism is likely due to the fact that the antibodies interfere with transport into the ER, and the prion protein is then rapidly cleared from the cytoplasm, inducing a loss-of-function defect, as described above [75].

One of three immunodominant B cell epitopes in the receptor binding domain of the spike protein spans the sequence from 439 to 478 (see [84], Figure 2). The last five amino acids in this sequence are YQAGS. This subsequence differs only by one amino acid from the sequence YQRGS in the globular C-terminal domain of the prion protein. This suggests that IgG antibodies produced in response to the mRNA vaccine could bind to the C-terminal domain and disable the prion protein from entering the ER, resulting in its clearance from the cytoplasm and inducing prion-like disease.

Interestingly, it has been shown experimentally that neutralizing antibodies are produced by convalescent patients in response to SARS-CoV-2 infection that binds to the YQAGS sequence. Wang et al. referred to this specific antibody as “XMA01,” and they promoted its use as a monoclonal antibody because of the stability of this region of the spike protein over multiple variants of concern, including Omicron [85]. However, there may be a risk of a CJD-like syndrome after treatment with these monoclonal antibodies, via molecular mimicry.

Relations of PrP^C^ and APP to phosphorylation pathways and beyond

Protein aggregation is common in some neurodegenerative diseases, such as AD, Parkinson’s Disease (PD), and Huntington’s Disease (HD). However, another common characteristic of prion and prion-like diseases is the improper conformation alignment of their disease-related proteins, i.e. PrP for prion diseases, tau and β-amyloid for AD and HD respectively, and α-synuclein for PD. The improper protein conformations are the tertiary structure alterations from α-helix to β-pleated sheets that then favorably follow the aggregation pathways which are thereafter resistant to proteasome degradation pathways [49]. In this regard, even slight modification in the amino acid terminus of proteins means an alteration in the N-degron recognition signaling for degradation [86].

Although PrPC participates in vitally important cellular functions (Table 1), the conformational conversion of PrPC to PrPSC is the hallmark to prion disease progression, and the prerequisite for this conversion is the expression and presence of PrPC. In the absence of endogenous PrPC there is an overwhelming resistance to the development of prion disease [87].

On the one hand, the role of normal isoform PrPC presence seems to be protective for cells, for example is the case where the suppression of PrP mRNA expression leads to the onset of premature aging processes [75]. On the other hand, the tissues that do not express PrPC are resistant to PrPSC toxicity. It is the infectious PrPSC that aggregates to form fibrils and the oligomers of these fibrils that are highly infectious and neurotoxic, and it is their relations to phosphorylation pathways that constitute the pathogenesis mechanisms of prion and prion-like diseases [15, 88-89]. Additionally, the intramolecular regions (tandem repeats) of tau protein strongly interact with the octapeptide repeats of wild-type PrP, and more strongly with the mutant types of PrPSC, to form strongly bound complexes [90]. This highlights the potential mutual involvement of both PrPC and tau proteins in the context of common pathogenic mechanisms causing prion disease, as well as tau-related neurodegeneration. The prion-like propagation that ensues also involves β-amyloid protein aggregation, which induces tauopathy as it is encountered in AD [88-89, 91].

Importantly, the acceleration of PrPSC formation through the cellular pathways just described drives forward, in a positive-feedback manner, the initiation and progression of tau-related pathology, including the production and aggregation of tau proteins. It is within this context that the events of inter-related neurodegenerative pathogenesis mechanisms transpire. Moreover, the advance and proliferation of misfolded PrP to an at-risk human organism’s neuronal tissues precedes the onset of neuro-pathogenesis disease mechanisms, suggesting that PrPC over-expression is a major contributor to the onset of prion and prion-like diseases [88].

Wip1 expression and the resolution of p38 MAPK activation

The formation of the PrPSC infectious isoform triggers a molecular cascade of neurotoxic events that involves the p38 MAPK pathway [15]. P38 MAP kinase phosphorylates and activates p53, a nuclear transcription factor that responds to stress signals, particularly DNA damage, and induces growth arrest, DNA repair, and apoptosis [92-93]. Autophagy inhibition can sustain p53 expression in an active state, accelerating the pathway toward apoptosis [94]. Wild-type p53-induced phosphatase 1 (Wip1) is a serine/threonine phosphatase, which plays an essential role in the resolution of the DNA damage response by downregulating p38-p53 signaling during the recovery phase [95]. Wip1 is overexpressed in many tumors [96-97] and under-expressed in neurons in association with neurodegenerative diseases such as amyotrophic lateral sclerosis (ALS) [98].

Stressors that induce the p38/MAPK response result in sustained phosphorylation of p53, which not only arrests the cell cycle but can also induce apoptosis when repair processes are overwhelmed with too many DNA double-strand breaks. By dephosphorylating several tumor suppressors, most notably p53, Wip1 inhibits apoptosis and promotes tumorigenesis, tumor progression, invasion, and metastasis [96].

Tumor cells are often somewhat reckless in proliferating even in the presence of DNA damage, which accelerates their mutation rate, whereas mature neurons are non-proliferating cells even in the absence of stressors. Because the base level of Wip1 is low in neurons, they are more vulnerable to apoptosis following p38/MAPK signaling, because the phosphorylated state is maintained for an extended period of time.

p53 plays an important role in neuronal apoptosis. A number of different stressors, including oxidative stress, DNA damage, metabolic impairments, and calcium overload, can cause a rapid increase in the synthesis of p53 in neurons. [99]. p53 upregulation leads to apoptosis in neurons that eventually results in symptoms of neurodegenerative disease, and agents that inhibit p53 may be an effective therapy for neurodegenerative disease [99].

Wip1 expression is controlled through a complicated regulatory process, which begins with p38/MAPK activation. Perhaps surprisingly, Wip1 transcription is upregulated by phosphorylated p53, simultaneous with the upregulation of many tumor suppressor genes, but its translation into protein is delayed. This is because miR-16 is also induced by p53 [100], and this microRNA suppresses translation and promotes clearance of Wip1 RNA [97]. As the repair process progresses, the level of miR-16 falls, such that Wip1 becomes functional only after a delay period, during which the neuron either recovers from the damage or undergoes apoptosis. As more and more neurons die, the symptoms of impaired cognition and memory start to become manifest [99].

Phosphorylation pathways: Wip1 expression and the role of p53

Experimental data strongly suggest that the p38 MAPK pathway is central to the development of neurodegeneration by infectious prions. The study conducted by C Fang et al., 2018 [15] utilized a specific neuronal culture system that distinguishes the cellular and molecular mechanisms by which prions cause damage in neural synapses. The authors used specific inhibitors against the three main families of MAPK, namely a) the extracellular signal-regulated kinases (ERKs), b) the Jun amino-terminal kinases (JNKs), and c) the p38 -stress activated protein kinases (SAPKs) in order to determine which of the distinct subfamilies of kinases are involved in the synaptic toxicity process caused by PrPSC. The authors concluded that the main kinases involved in dendritic spine toxicity were those of p38 MAPK subfamily, and, in particular, the p38α isoform. Furthermore, a p38 MAPK inhibitor, after 24 h of being added to the culture, was able to completely reverse the initial synaptic toxicity effects caused by PrPSC. Moreover, the authors also used a genetic method of suppressing the p38 MAPK activation cascade by culturing a hippocampal neuron cell line which is heterozygous for p38α MAPK (T180A/Y182F), p38AF. This dominant negative mutant cell line was also protected from PrPSC synaptic neurotoxicity in a way comparable to the effect of the p38α inhibitor. In a relevant study, a double mutation in the activation site of p38AF protein, at the sites of Thr180 and Tyr182, inhibits the phosphorylation of the p38 molecule by other kinases. Also in this study, the heterozygous mice for the p38AF (+/-) allele show a marked reduction in a) p38-related signaling and b) the expression of age-produced cell cycle inhibitors [101].

Additionally, the mutated p38AF animals showed increased proliferation and regeneration of pancreatic islets, amongst other organs. Overall, in this study, the p38AF mutated animals expressing the defective isoform of p38α AF possessed a resistant mechanism that alleviated synaptic toxicity caused by PrPSC (spine degeneration), thereby bypassing the mechanism of PrPSC activation of a localized p38-mediated signaling cascade that leads to dendritic spine retraction [15, 101]. Importantly, the p38AF, Wip1 deficient mice showed a reduction in their cellular proliferation capacity. By contrast, the animals that showed Wip1 overexpression retained their cellular capacity of induced regeneration.

The Wip1 deactivation observed during the natural aging of p38AF mutated animals, concurrent with their genetically induced loss of p38 MAPK activation, is highly relevant to PrPSC propagation by the SARS-CoV-2 spike protein. It shows that spike-protein-induced neurotoxicity, as explained in more detail below, would be predicted to be age-related. The p38 MAPK pathway, being inactivated in the p38AF mutated animals, did not influence the Wip1 activity. Thus, these two distinct but inter-related phosphorylation pathways are being concurrently yet independently inactivated due to aging [101].

Under normal circumstances, the p38 MAPK pathway is activated (phosphorylated) by the upstream induction of TLR activation via Myeloid Differentiation primary response (MyD88 adapter protein), and downstream by the TGFβ-Activated Kinase 1 (TAK1), which becomes active through auto-phosphorylation [34]. Moreover, the MyD88 induction involves both TLR2 and TLR4 activation (via the CD14 receptor), with the final outcome being the promotion of the NF-κB response [102]. However, it is through the TLR4 activation and subsequent p38 MAPK pathway follow-up of phosphorylation events that the inflammatory response of Il-1β, Il-6 and TNF-α is being presented. The activation of IRAK4 phosphorylation by the SARS-CoV-2 spike protein has been shown to be induced by both TLR2 and TLR4 activation that subsequently produces a similar interleukin-mediated inflammatory response in human macrophages [103]. Furthermore, the same pattern of TLR2 and TLR4 activation to produce NF-κB and the interleukin-mediated inflammatory response is also occurring in injured or damaged microglia and astrocytes [104].

Particularly, the TLR4 receptor serves as an upstream regulator of Wip1 phosphatase in cells in the nervous system [105-106]. In astrocytes, Wip1 expression provides a negative feedback loop in response to the activation of the NF-κB response. In brief, although TLR4 activation led to an increase in Wip1 and phospho-NF-κB-p65 expressions in LPS-stimulated primary astrocytes, the expression of p65 was further increased when the expression of Wip1 was deactivated [105]. Similarly to the LPS-induced activation of TLR4 in human monocytes, the SARS-CoV-2 spike protein induces a comparable interleukin (IL-1β) response also via activating TLR4 [107]. Similar induction of IL-1β was noticed in a differentiated neutrophil cell line that expressed TLR4 following spike protein exposure. Also, the spike protein was able to induce an IL-1β response in various murine macrophage cell lines, specifically due to TLR4 expression.

In conditions of brain injury, the expression of Wip1 in the nervous tissue prevents inflammation by inhibiting microglial and macrophage accumulation [108]. In murine and human macrophages, the SARS-CoV-2 spike protein, and specifically the S1 subunit of the trimer, activates NF-κB and c-Jun N-terminal kinase (JNK) pathways specifically via TLR4 activation [14]. Additionally, in microglial cells, which are a specialized macrophage type of cell in the brain, the induced spike protein neuroinflammation via TLR4 activation includes sustained NF-κB activation, suggesting that Wip1 expression is weak and/or delayed [12]. ROS-dependent activation of JNK causes p53 to robustly induce apoptosis, and this is considered to be a feature in tumor cells, but it may be worrisome when neurons are exposed to JNK activation in the context of highly phosphorylated p53 [109].

Thus, although downregulation of Wip1 expression is positively correlated with better recovery from sepsis by activating neutrophil migration and thus enhancing antimicrobial activity at the point of infection [110], the loss of Wip1 expression in the nervous system can be viewed as being tightly correlated with increased inflammation by uncontrolled, p65-dependent, induction of NF-κB signaling. In that regard, the increased p53 activity can be viewed as a normal function to promote apoptosis in order to prevent the emergence and persistence of cells with damaged genomes [111].

Wip1 activity and regulation of expression

Wip1 phosphatase is a critical protein regulating the DNA damage repair processes. Following DNA damage repair, the homeostatic mechanisms of the cell require Wip1 activity to release cells from cell cycle arrest, by dephosphorylating and thus inactivating p53, p38 MAPK, ataxia telangiectasia mutated (ATM), and other stress-induced proteins (for review of Wip1 targets, refer to J. Lowe et al., 2013) [112]. With p53 no longer inducing cell cycle arrest, the cell is able to return to its original unphosphorylated state.

The PPM1D/Wip1 gene was originally discovered as a p53-induced gene. However, it has since been discovered that its expression depends also on many other stress-induced transcription factors apart from p53. Mainly, its product, Wip1, provides a negative feedback loop for the activity of many DNA repair factors including the dephosphorylation, and thus inactivation, of histone 2HX-γ (H2AX-γ), and p53 regulating (inhibitor) molecules [95, 113].

Furthermore, the over-expression of Wip1 negatively regulates the NF-κB response by reducing TNF-α induced phosphorylation of the serine 536 of p65 and reducing its binding with p300. The effects of Wip1 activity on the inhibition of NF-κB and chromatin remodeling are independent of p38 MAPK pathway activation [114]. Notably, Wip1 expression is decreased when NF-κB activity is inhibited in primary astrocytes, indicating a positive regulation of NF-κB on the PPM1D gene and, further on, the neuroinflammatory regulation by Wip1 and NF-κB inhibition [105].

Wip1 expression is reduced during neutrophil activation and is directly inhibited by the increase of microRNA-16 expression which targets its 3’ untranslated region and thus regulates post-transcriptionally Wip1 translation. Finally, the TLR4 ligands, and the activation of inflammatory cytokines, downregulate Wip1 expression via the activation of microRNA-16 by p38 MAPK and NF-κB [97, 115].

Regulation of human prion protein and β-amyloid genes

The PRNP gene located in chromosome 20 in humans codes for PrPC in the central nervous system and several other tissues [50]. This is a highly conserved housekeeping gene, and it is subject to many transcription factors functioning in its promoter and thus regulating its expression. Amongst many others, putative sequences for transcriptional activation by activator protein 1 (AP-1), SP1 and SP2 (members of the SP/KLF family of transcription factors) have been identified as PRNP promoters.

Importantly, a short GC-rich region is located upstream from the PRNP gene promoter. These GC-rich regions have the potential to form G-quadruplex (G4) structures and therefore regulate gene disease-related expression, as they are subject to favorable binding by p53. The binding of p53 to GC regions forming G4s has been shown to initiate a series of cellular effects related to disease [116-117]. Furthermore, it has been shown that the PRNP promoter region harbors a sequence matching the binding sequence of p53. p53 binds directly to the suspected sequence, behaving as a potent PrPC transcriptional activator and enhancer of its mRNA expression [118]. In summary, p53 causes an increased expression of PrPC.

RNA translational regulation is considered an important contributor to PrPC conversion to infectious PrPSC. Beyond DNA, it has been shown that the messenger RNA of PrPC contains five naturally existing consecutive regions forming G4s that are susceptible to G4 binding ligands [119]. In this respect, p53 can be regarded as an RNA chaperone that is able to facilitate the folding of G4s and hence stabilize their structure [120]. G4s in 5’-untranslated mRNA regions are found in multiple neurodegenerative diseases and have been shown to inhibit translation and initiate cap-independent translation [121].

The amyloid precursor protein (APP) gene coding for the APP in humans is located on chromosome 21. Viewing its promoter sequence, it can be designated as a housekeeping gene like the PRNP gene. APP shares some important promoter sequences with PRNP like AP-1 and Sp1, amongst many others, which however differ from the sequences in the PRNP promoter. This suggests that both genes can be partly transactivated by the activity of common transcription factors [122].

APP mRNA is expressed in a variety of tissues, including muscle, the immune system, and many organs such as the thymus, pancreas, kidneys, the lung and others, in addition to its active expression in the nervous system. However, different variants of APP are cell-type specific in their expression [123].

The variants of APP include APP-like protein-1 (APPL1 gene located on chromosome 21) and APPL2 (APPL2 gene located on chromosome 11), which are both type 1 transmembrane proteins with similar structure and topology. Only APP itself, however, contains the Aβ sequence. The fibrillary form of Aβ (40-42 amino acids), found and constituting the primary source of plaques in the brains of patients suffering from AD and Down syndrome, originates only from APP proteolysis. The full length of human APP sustains proteolysis mainly via the α,β,γ-secretases. The derived amino acid sequence of Aβ results from the β-site APP cleaving enzyme 1 (BACE-1) or else β-secretase cleavage yielding APPsβ and APPCTFβ (βAPP) fragments of APP. Thereafter, the cleavage of γ-secretase on βAPP finally yields Aβ and the APP intracellular domain (AIDC) fragments (for details see [124]). Moreover, the AIDC fragment is also produced by α-secretase and subsequent γ-secretase activity.

γ-Secretase is also called presenilin-dependent γ-secretase, since it encompasses presenilin (PS) transmembrane proteins in its catalytic subunit (PS1 or PS2) [125]. In this respect, it has been established that γ-secretase/presenilin-dependent generation of AIDC operates as a transcriptional activator of p53, increasing p53 activity and triggering p53-associated cell death. Moreover, mutations in transcription factor Sp1 increase the p53 activity in vitro and in the brains of patients affected with familial Alzheimer’s disease (FAD) [118]. Mutations on Sp1 are considered a causative factor for FAD.

The tumor suppressor p53, once generated through the γ-secretase/presenilin-dependent transcriptional activation of the TP53 gene by AIDC bound to Fe65 and Tip60 cofactors, then acts on the promoter of PrPC and induces the expression of PrPC mRNA. The p53, γ-secretase/presenilin-dependent transactivation of PrPC expression is abolished in a p53-deficient environment. Thus, it is ultimately the PSs (PS1 or PS2) that exert rate-limiting control over PrPC expression through their ability to generate AIDC. Finally, βAPP overexpression increases PrPC expression, whereas βAPP depletion results in lower PrPC expression, in both in vitro and in vivo experiments, indicating also the controlling role of BACE-1 activity over PrPC expression [117]. Thus, the metabolism of APP that produces amyloidogenic products also induces increased production of PrPC.

The fine balance between autophagy and proteasome degradation in relation to neurodegeneration

A common characteristic of neurodegenerative diseases is a severe disturbance of protein homeostasis. Impaired clearance of misfolded proteins via autophagy/lysosomal degradation results in their accumulation within the cytoplasm [126].

p53 has multi-functional roles in macro-autophagy (hereafter termed autophagy), a state where the cell suppresses cellular regeneration and consumes/recycles intracellularly its constituents to maintain homeostasis and survival during starvation. Autophagy and p53 exhibit reciprocal functional interactions. p53 operates within a negative feedback loop with the process of autophagy: as p53 activity increases, autophagy is activated within the cell. With increased autophagy, negative feedback suppresses the activity of p53 [127]. During autophagy activation, the intracellular components are delivered to lysosomes for further degradation via both macro- and micro-autophagy pathways, as described in detail by Barbosa et al. [128].

The degradation of misfolded proteins is managed by two interrelated pathways: the ubiquitin-proteasome system (UPS) and macroautophagy (also referred to simply as "autophagy"). Sequestosome-1, also known as the ubiquitin-binding protein p62, plays a critical role in both pathways. p62 captures and presents ubiquitinated cargos for autophagy [129]. Decreased levels of p62 are linked to many neurodegenerative diseases [130]. Oxidative damage to the p62 promoter decreases p62 promoter activity, reducing the expression of p62, and therefore impairing autophagy. Its promoter is particularly rich in guanines that are especially susceptible to oxidative damage [130]. The inhibition of proteasome degradation results in impaired clearing of substrates such as p53 and β-catenin, and this results in a twofold increase in their levels in cellular models. These same elevated levels are reached when the UPS is blocked, even when autophagy is not inhibited.

Since many UPS substrates such as p53 mediate toxicity, impaired removal of such regulatory proteins via autophagy is recognized as a prerequisite for many severe disease states, such as in the case of prion disease, solely due to the intracellular increase of aggregation-prone proteins [94]. Furthermore, the activation of autophagic mechanisms is lowered with advancing age, constituting an extra parameter for susceptibility to neurodegenerative disease due to autophagic inhibition [128].

With respect to the development of prion disease, specific in vitro and in vivo models have shown that reduced gene expression of p38 MAPK facilitated the clearance of BACE-1 through lysosomal degradation. This resulted in a decrease in the intracellular level and activity of BACE-1, and, ultimately, lower Aβ levels in the mouse brain, associated with enhanced autophagic mechanisms. Thus, the knockdown of p38 MAPK in neurons reduces Aβ generation and decreases Aβ load by promoting macroautophagy. Moreover, in a separate experiment, the authors treated human cells with an autophagy inhibitor, and this also increased BACE-1 protein levels and even abolished the p38-MAPK knockdown-induced decrease of BACE-1 protein. These findings demonstrate that p38 MAPK activation and autophagy inhibition are vital for the progression of prion disease [131].

In relation to SARS-CoV-2 spike protein being a toxic factor for prion disease, these findings are of major importance, since infectious prions are shown to activate the p38 MAPK signaling response. In an equal fashion, and in a dose-dependent manner, the S1 subunit of the spike protein has been shown to a) increase p38 MAPK protein levels, b) increase phosphorylated p38 levels, c) increase the inflammatory cytokines IL-6 and TNF-α, amongst others, d) increase TLR2/4 protein levels and thus signaling, and e) increase NF-κB protein activity and binding to provide transcriptional control over the established neuroinflammation in S1-induced BV2 microglia [12, 15].

SARS-CoV-2 spike protein suppresses DUSPs to further induce neurodegeneration

In addition to Wip1, dual-specificity phosphatases [DUSPs] are a large heterogeneous group of protein phosphatases that can dephosphorylate serine, threonine, and tyrosine residues on a large number of proteins. Many of the proteins that they dephosphorylate are part of the MAPK cascade, and therefore they can be effective to turn off MAPK activation and resolve an inflammatory response [132].

Because DUSP genes, especially DUSP1 protein, are negative regulators of p38 MAPK signaling, their reduction under TLR4 signaling will sustain the activation of both p38 MAPK and c-Jun NH2 terminal kinase (JNK) pathways [106, 133-134].

As we have seen, several multidisciplinary studies provide evidence of the activation of TLR2/4 signaling by the SARS-CoV-2 spike protein [12-13, 38-39, 107]. Especially in nerve cells, the S1 subunit of the spike protein activates p38 MAPK and NF-κB through upregulation of expression and activation of TLR4 pattern recognition receptor [12]. Furthermore, exposure of human macrophages to the spike protein activates the phosphorylation of IRAK4 and the subsequent p38 MAPK and JNK pathways, and resulting in the suppression of autophagy [103].

Notably, SARS-CoV-2 infection and subsequent cleavage of the spike protein by the transmembrane protease/serine subfamily 2 (TMPRSS2) / p38 MAPK pathway activates MAPK phosphorylation and NF-κB signaling by reducing the transcriptional activation of DUSP1 and DUSP5 [135]. This is a unique property of SARS-CoV-2 compared to all other coronaviruses. Moreover, p53 has been shown to enhance the post-transcriptional maturation of miR-16 [100], and, as we have seen, miR-16 has been shown to downregulate the expression of Wip1 [97].

Thus, the Wip1 and DUSP inhibitory activity upon p53, p38 MAPK, and ATM will both be attenuated in the presence of the spike protein. As a consequence, there will be sustained production of inflammatory cytokines, and an increased tendency towards cellular senescence and apoptosis [112]. β-Amyloid (Aβ) production occurs in various cell types and in many organs [124, 136]. However, in cells orchestrating simultaneous Aβ / AICD production and PrPC expression, i.e., neurons, the spike-protein-induced impairment of the phosphatase pathways will have deleterious effects, with significant implications for cellular neurotoxicity [137-138].

The excess phosphorylated p53 from the suppression of Wip1 and DUSP dephosphorylation activities acts as a transcriptional activator of the prion protein promoter to produce an excess of PrPC, creating an environment for prion disease development. Since presenilin-dependent γ-secretase works in concert with p53 by enhancing its expression through producing AICD and Aβ, it thus worsens the preconditioning of spike-protein-induced neurotoxicity in this system. Furthermore, the increased expression of transcription factor AP-1 by the phosphorylated c-Jun triggers the promoters of APP and PRNP for further transcriptional activation [118, 136].

**Figure 1:**
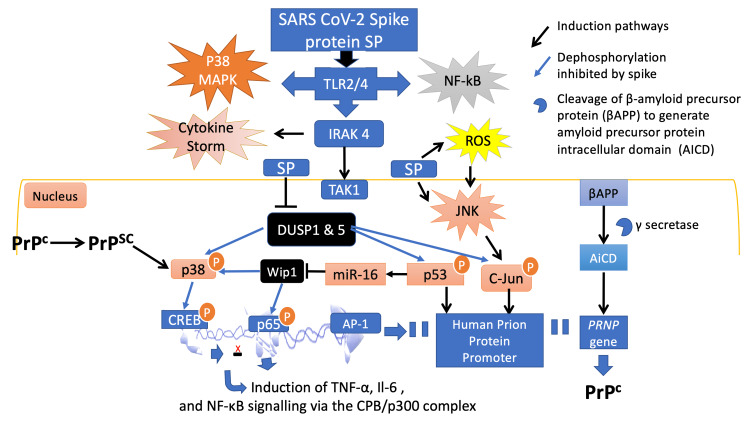
The phosphorylation pathway induced by SARS-CoV-2 spike protein leads to prion disease. The spike protein activates TLR4 signaling to induce p38 MAPK and NF-κB. Moreover, the spike protein also stimulates IRAK4 signaling to induce p38 MAPK, NF-κB and cytokine storm and inhibits DUSPs and Wip1, causing sustained p53 expression. Wip1 deficiency caused by JNK-microRNA-16 activation leads to diminished p53 deactivation and thus, transcriptional activation of the human PrP promoter. This leads to increased accumulation of PrP^C ^and to induction of IL-6 and TNF-α cytokines through p38/CREB, and p65/NF-κB activation. Accumulation of PrP^C^ is a predisposing factor for the conformational alteration to PrP^SC^ and therefore prion and prion-like diseases. PrP^SC^, once formed, will further enhance p38 MAPK activation. Adapted from: Refs. [1, 12, 15, 38, 103, 105-107, 115, 118, 133, 142].

Common transcription factor activation located in both APP and PRNP promoters, such as by the selective promoter factor 1 (SP1), happens during the inflammatory response in the AD brain. Among many other important roles, AP-1 regulates the transcription of BACE-1, and the tau protein subsequently promotes the development of neurotoxicity [139-141]. The condition can be described as ‘Wip1 and DUSP deficiency-p53 mediated induction of prion and prion-like disease induced by the SARS-CoV-2 spike glycoprotein’ and is illustrated in Figure 1.

Relation of SARS-CoV-2 spike-protein-induced neurotoxicity to age and the inhibition of autophagy

The relationship between age and the reduced cellular capability for autophagy, in combination with p53 accumulation during autophagy inhibition, constitutes the proposed model of spike-protein-induced neurotoxicity presented in Figure 1. In this model, pathogenesis is augmented by a) aging, which leads to impaired autophagy, and b) p53 accumulation, due to the inhibition of the UPS system for degradation [127-128].

Under autophagy inhibition and p38 MAPK activation, a detrimental cascade of events ensues: Wip1 deactivation, and, hence, inhibition of p53 dephosphorylation, concurrent with BACE-1 activation, both promote AIDC positive regulation of the TP53 gene and the p53-dependent transcriptional activation of the PRNP gene. These events set the stage for the cascade of cellular events leading to prion protein aggregation and subsequent pathologies.

The release of p53 from dephosphorylation by DUSP1 or Wip1 drives the neuron towards the onset of prion and protein folding diseases and establishes the cellular circumstances whereby the SARS-CoV-2 spike protein can play a central role in creating neurotoxicity and predisposing exposed individuals toward neurodegeneration. However, this process is age-dependent, and it is related to the cellular ability to induce autophagy. Although the clear relationship between PrPC and PrPSC formation has not yet been established, the generation of infectious prions is clearly related to the induction of the p38 MAPK pathway, which is also induced by the spike protein in conjunction with JNK in several ways.

**Figure 2:**
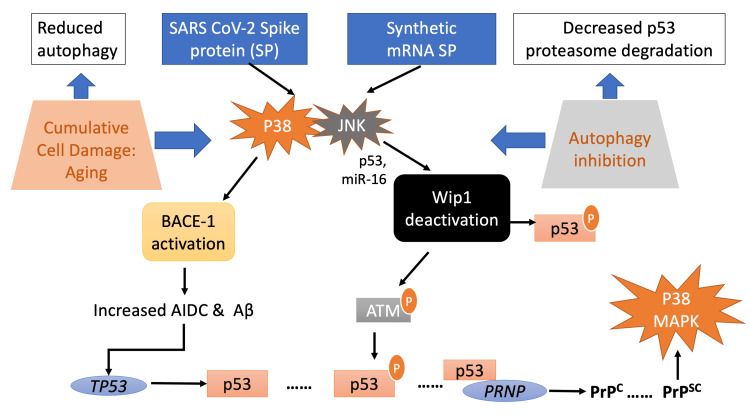
The SARS-CoV-2 spike protein neurotoxicity dependence on age and inhibition of autophagy. The ability to induce autophagy is age dependent. Autophagy is inhibited in part through DNA damage to the sequestosome p62 promoter, caused by oxidative stress. The activation of p38 MAPK and JNK pathways by the spike protein in nerve cells leads to BACE-1 activation and, through JNK-mediated Wip1 deactivation, increases activated (phosphorylated) p53. The release of AIDC via APP metabolism further enhances TP53 transcriptional activation and hence p53 expression. Free P53 can be further phosphorylated by ATM (being active through JNK-dependent microRNA-16 Wip1 inhibition). The overall process leads to the accumulation of levels and expression of PrP^C^. Conformational alteration of PrP^C^ to PrP^SC^ induces the activation of p38 MAPK, constituting the whole age-dependent process. Adapted from: Refs. [12, 15, 94, 112, 115, 118, 127-128, 131, 133, 143].

Figure 2 shows the potential mechanisms of the SARS-CoV-2 spike protein, derived either from natural infection or from synthetic mRNAs coding for SP, that induce prion and prion-like disease. The spike-protein-induced neurotoxicity mechanism depends on a) the age of the spike protein recipient and b) the impairment of suppression of prion disease through macro-autophagy [1, 15, 112, 131, 103].

## Conclusions

In this article, we have reviewed the research literature on the spike protein-related processes that lead to the development of neurodegenerative disease, in the context of several recent papers reporting on the observed mechanisms of toxicity. We were initially motivated by the observation that COVID-19 patients often suffer from long-term sequelae that include cognitive impairment -- so-called long-haul COVID disease. There is also a post-vaccination syndrome that strongly resembles long COVID.

Central to the promotion of prion and prion-like disease is the induction of γ-secretase metabolism of the APP sequence, which, through BACE-1, yields the AIDC sequence, a highly potent transcriptional activator of the TP53 gene. This disease-prone metabolic state is induced through p38 MAPK activation in neurons. Therefore, the SARS-CoV-2 spike protein can be a re-enforcing toxicity factor, since it induces both p38 MAPK and JNK activation which subsequently will provide a surplus of activated p53. The activation of p53 is potentially further enforced through concurrent Wip1 deactivation by JNK-p53-induced miR-16 expression. Decreased degradation of p53 via the UPS and autophagy due to oxidative damage to the p62 promoter further enhances the risk of induction of neuronal apoptosis. An autoimmune attack on neurons due to molecular mimicry likely plays a contributory role.

We propose that age-related impairments in autophagy may predispose towards increased risk to cognitive issues associated with the ability of the spike protein to behave as a prion-like protein, triggering misfolding of PrP and other amyloidogenic proteins. The spike protein has been shown to induce an inflammatory response in microglia, which can lead to oxidative stress and DNA damage. Through MAPK activation via TLR4 receptors, as well as JNK activation, the spike protein can be expected to suppress key phosphatases that normally would restore cellular homeostasis following p53 activation via MAPK. Sustained p53 phosphorylation in neurons can induce PrPC conversion to PrPSC. The precipitation of misfolded PrP into fibrils causes a loss-of-function pathology, and subsequent catastrophic autophagy failure ultimately leads to programmed cell death (apoptosis) and resulting neurological symptoms and accelerated senescence.

Our work has important implications for public policy given the continued widespread application of COVID-19 vaccines. If the spike protein conceivably could contribute to future neurodegenerative diseases, then the risk-benefit calculation for mass indiscriminate vaccination should be re-examined. If the arguments presented here are found to be true, the vaccinated population has already been subjected to a great deal of harm.

*Note*: A previous version of this article was posted to the Authorea preprint server on November 16, 2022.
